# Lung function in relation to brain aging and cognitive transitions in older adults: A population‐based cohort study

**DOI:** 10.1002/alz.14079

**Published:** 2024-07-05

**Authors:** Giulia Grande, Yuanjing Li, Caterina Trevisan, Debora Rizzuto, Grégoria Kalpouzos, Mozhu Ding, Erika J Laukka, Tom Bellander, Laura Fratiglioni, Chengxuan Qiu

**Affiliations:** ^1^ Aging Research Center Department of Neurobiology Care Sciences and Society Karolinska Institutet and Stockholm University Stockholm Sweden; ^2^ Stockholm Gerontology Research Centre Stockholm Sweden; ^3^ Department of Medical Sciences University of Ferrara Ferrara Italy; ^4^ Unit of Epidemiology Institute of Environmental Medicine Karolinska Institutet Stockholm Sweden

**Keywords:** brain aging, dementia, mild cognitive impairment, peak expiratory flow, population‐based study

## Abstract

**BACKGROUND:**

We investigated the association of peak expiratory flow (PEF) with dementia; cognitive impairment, no dementia (CIND); and transition from CIND to dementia, and possible underlying neuropathological mechanisms.

**METHODS:**

A population‐based cohort of adults aged 60+ was followed over 15 years to detect dementia (Diagnostic and Statistical Manual of Mental Disorders, 4th edition criteria), CIND (assessed through a cognitive battery), and progression from CIND to dementia, in relation to baseline PEF observations. A subsample (*n* = 462) had 6‐year follow‐up data on brain magnetic resonance imaging markers of neurodegeneration and small vessel disease.

**RESULTS:**

In fully adjusted models, poor PEF performance (< 10th vs. ≥ 80th percentile) was associated with increased hazards for dementia (hazard ratio [HR] = 1.89; 95% confidence interval [CI] = 1.23–2.92) and CIND (HR = 1.55; 95% CI = 1.01–2.38) and CIND progression to dementia, although not statistically significantly (HR = 2.44; 95% CI = 0.78–6.88). People with poor PEF also experienced the fastest ventricular enlargement (β coefficient = 0.67 mL/year; 95% CI = 0.13–1.21) and had the highest likelihood of developing lacunes (odds ratio = 5.05; 95% CI = 1.01–25.23).

**DISCUSSION:**

Poor lung function contributes to cognitive deterioration possibly through accelerated brain atrophy and microvascular damage.

**Highlights:**

- Poor lung function increased the risk of dementia and mild cognitive impairment (MCI).
- Poor lung function accelerated the progression from MCI to dementia.
- Poor lung function was linked to brain microvascular damage and global brain atrophy.

## 1 BACKGROUND

Along with aging, lung function tends gradually to decline as a result of several structural, physiological, and immunological changes.^1^ Poor lung function has been associated with a number of adverse health‐related outcomes, including disability and shorter survival.^2^ Notably, accumulating evidence has also linked poor pulmonary function with cognitive impairment and dementia in old age.^3^, ^4^ A meta‐analysis pooling data from four longitudinal studies found that poor pulmonary function as assessed with peak expiratory flow (PEF) was linked with an 80% increased risk of dementia.^4^

Given the observed association with overt dementia, it is plausible to hypothesize that poor pulmonary function may be linked with the prodromal stages of dementia, like mild cognitive impairment (MCI).^5^ A meta‐analysis including eight longitudinal studies examined the association between pulmonary function and cognitive performance in aging and found consistent but weak results.^6^ In addition, only one study has so far suggested that poor lung function accelerates the progression from MCI to dementia.^7^

Low PEF may capture multiple environmental insults across the lifespan, including smoking, air pollution, lung diseases, but also physical vulnerability, all of which have been linked, in turn, to cognitive dysfunction and dementia.^6^, ^8^, ^9^ A recent study from our group including almost 3000 Swedish individuals reported that PEF was a marker of general robustness in older adults, and a reduced PEF was associated with frailty development.^10^ Whether this observation is valid also when looking at cognitive outcomes is still unknown.

The neuropathological mechanisms underlying the associations of lung function with cognitive phenotypes are yet poorly understood. Cross‐sectional population‐based studies in older people have found limited expiratory function to be associated with lower volumes of total brain tissue and the hippocampus, higher volume of white matter hyperintensities (WMH), and higher prevalence of lacunes.^11^, ^12^, ^13^, ^14^ However, there is a lack of understanding about the longitudinal association of limited expiratory flow with pathological brain aging, as previously only one study has investigated the link between forced expiratory volume (FEV1) and brain imaging markers such as ventricular volume, brain infarcts, and WMHs.^15^

In this population‐based study, we aimed to investigate the association of PEF with dementia; cognitive impairment, no dementia (CIND); and progression from CIND to dementia in older adults and further to explore the neuropathological mechanisms underlying these associations. We hypothesized that low PEF is associated with a higher risk of cognitive decline and transition from CIND and dementia and of a faster accumulation of structural brain damage.

## 2 METHODS

### 2.1 Study population

We used data from the Swedish National Study on Aging and Care in Kungsholmen (SNAC‐K, 2001–2004 to 2016–2019), an ongoing population‐based longitudinal study, as previously described.^16^ In brief, at baseline (2001–2004), eligible participants were aged 60+ years and lived in the Kungsholmen district in central Stockholm. During the baseline assessment, 3363 (response rate 73.3%) participants were examined. Since then, participants have been followed up regularly: every 6 years for the young‐old cohorts (age < 78 years) and every 3 years for older cohorts (age 78+ years).

For the current study, we identified three analytical samples within the SNAC‐K cohort to address our aims: In analytical sample 1, we assessed the association between PEF and incident dementia, thus we included a dementia‐free population with a Mini‐Mental State Examination (MMSE) ≥ 27 (*N* = 2747). Of these, 257 individuals had missing data on PEF, and 165 were lost to follow‐up, so we ended with a final sample of 2323 dementia‐free participants for this analysis. In analytical sample 2, we aimed to assess the association between PEF and incident CIND; thus, the sample consisted of 1908 cognitively intact individuals, that is, persons who were free from dementia and CIND at baseline; of these, we further excluded those with missing data in PEF (*N* = 129) and those with no follow‐up data (*N* = 308), obtaining a final sample of 1471 individuals with complete data. In analytical sample 3, we aimed to assess the association between PEF and progression from CIND to dementia; thus, we included 495 participants with CIND and an MMSE score ≥ 27; of them, we excluded those with missing data on PEF (*N* = 52) and those with no follow‐up data (*N* = 23), ending with a final sample of 420 participants with complete data. Figure S1 in supporting information shows the flowchart of study participants for the three analytical samples.

Overall, those with missing data at baseline were older, more likely to be female, less educated, and with a greater number of chronic diseases compared to those with no missing data. Conversely, those with no follow‐up data were more likely to be younger, more educated, and with fewer chronic diseases as those who had at least one follow‐up assessment.

In addition, a subsample of 555 non‐institutionalized, non‐disabled, and non‐demented participants from SNAC‐K undertook structural brain magnetic resonance imaging (MRI) examination at baseline (2001–2004), and follow‐up MRI scans were performed in 2004 through 2007 and 2007 through 2010. Of the 555 baseline participants, 93 individuals were excluded due to suboptimal quality or incomplete baseline MRI sequences or brain disorders (e.g., brain infarcts, brain tumors, and arachnoid cysts; *n* = 45) and missing data on PEF (*n* = 48), leaving 462 dementia‐free people with available brain MRI markers. Of these, 299 (64.7%) had at least one follow‐up MRI examination.

The Regional Ethical Review Board in Stockholm, Sweden, approved the protocols of the SNAC‐K study. All participants, or next of kin for cognitively impaired individuals, provided written informed consent.

The results of this study are reported following the Strengthening the Reporting of Observational Studies in Epidemiology recommendations.

### 2.2 Data collection

Data were collected at our research center in accordance with standard procedures. Trained staff collected data via face‐to‐face interviews by nurses, clinical examinations by physicians, neuropsychological testing by psychologists, and laboratory tests. Home visits were conducted for those who agreed to participate but who were unable to come to the research center.

We collected data on demographics (e.g., age, sex, and education), lifestyles (e.g., smoking), health history (e.g., asthma, diabetes, and cardiovascular disease), and cognitive function. Education was measured as the highest level of formal education and categorized as elementary school, high school, and university or above. Smoking was categorized as current, former, or never smoking. The MMSE was used to measure global cognition. Details on the definition and classifications of chronic diseases are reported elsewhere.^17^ Briefly, the examining physicians collected information on health conditions via physical examination and considering medical history, the use of medications, self‐reported health problems, and/or proxy to identify specific health conditions. All diagnoses of health conditions were coded according to the International Classification of Diseases 10th revision (ICD‐10) and classified into 60 chronic disease categories in accordance with a clinically driven methodology. For the current study, both the total number of chronic diseases and the presence of asthma and chronic obstructive pulmonary diseases (COPD) were considered. In addition, we considered diabetes, atrial fibrillation, ischemic heart diseases, and cerebrovascular diseases in sensitivity analyses. Information on inhaled bronchodilator use (Anatomical Therapeutic Chemical codes: R03BA, R03AK, R03AL08, and R03AL09) was recorded. Body mass index (BMI) was obtained by dividing weight by squared height (kg/m^2^). A BMI < 18.5 kg/m^2^ was considered a proxy of undernutrition.^18^ Walking speed was assessed by asking the participants to walk at the usual pace over 6 or 2.4 m (if the individual reported walking slowly or if space was restricted). When the participant was unable to walk, a value of zero was given. The date and cause of death were obtained from the Swedish Cause of Death Registry.

**RESEARCH IN CONTEXT**

1. **Systematic review**: We searched PubMed and Web of Science for studies investigating the relationship of lung function with cognitive function and dementia. We found two meta‐analyses reporting that poor pulmonary function increased dementia risk. Only one study examined the association of poor lung function with progression from mild cognitive impairment (MCI) to dementia. In addition, only one study has investigated the longitudinal link between forced expiratory volume and brain imaging markers.
2. **Interpretation**: Impaired pulmonary function is associated with an accelerated transition from normal cognition to MCI and dementia. We further revealed that poor lung function is associated with cerebral microvascular damage and accelerated global brain atrophy, which might partially explain the association of poor lung function with cognitive deterioration.
3. **Future directions**: Longitudinal studies involving neuroimaging and blood Alzheimer's disease biomarkers may help further elucidate the biological mechanisms underlying poor pulmonary function and cognitive aging, which might open new research avenues for the prevention and treatment of dementia.

### 2.3 Definition of CIND

CIND was operationalized using the neuropsychological test battery as an objective impairment in cognition that did not meet the diagnostic criteria for dementia. At each wave, five cognitive domains were assessed: executive function (Trail‐Making Test, Part B), episodic memory (free recall), visuospatial abilities (mental rotations), language (category and letter fluency), and perceptual speed (digit cancellation and pattern comparison).^19^ To calculate age‐specific cognitive norms, we first standardized the raw test scores into *z* scores using the baseline age‐specific mean and standard deviation (SD). When more than one cognitive test was available, we created a composite score for the cognitive domain by averaging *z* scores of tests for that domain. Participants were identified as having CIND if they scored more than 1.5 SDs below the age‐specific mean in at least one cognitive domain. The same procedure was used to identify CIND at follow‐up visits, using the baseline means and SD.

### 2.4 Diagnosis of dementia

The diagnosis of clinical dementia was made following the criteria outlined in the Diagnostic and Statistical Manual of Mental Disorders, Fourth Edition (DSM‐IV), following a three‐step procedure. First, a preliminary diagnosis was made by the examining physician, followed by a second preliminary diagnosis by a reviewing physician who was also involved in the data collection. In the case of disagreement between the first and the second diagnoses, the final diagnosis was made by senior neurologists who were external to the data collection. To ascertain possible dementia diagnoses among those who died between the follow‐up examinations, clinical charts of those who died were collected with their death certificates and examined by the same physicians.

### 2.5 Assessment of peak expiratory flow

We measured PEF using a mini‐Wright peak flow meter (Airmed Clement Clarke International), a hand‐held device for assessing the maximal speed of expiration of an individual. Participants were instructed to breathe in as deeply as possible and then blow as hard as they could into the instrument. The test was repeated three times, and the highest value, expressed as liters/minute, was considered in the analyses.^20^ In accordance with previous studies and considering the physiological decline of PEF observed with aging and its interindividual variability by age, sex, and body height, we operationalized the PEF variable following a two‐step procedure. First, we estimated expected PEF values through sex‐specific predictive equations that included age and body height obtained considering a comparatively healthy subsample of the same population (i.e., people who had never smoked and had no diagnosis of respiratory disorders, cardiovascular diseases, or cancer).^21^ Second, we computed PEF standardized residual (SR) percentiles from a normalization of the ratio (measured‒predicted PEF)/(standard deviation of the residuals), where SR = 0 corresponds to the 50th percentile.^22^ SR percentiles were used as a continuous variable per 10th SR percentile decrease. Moreover, to evaluate the possible dose–response effect in the tested association and to facilitate comparison of our findings with the literature, we categorized PEF SR percentiles into four groups (< 10th, 10th–49th, 50th–79th, and ≥ 80th percentiles) considering standard cut‐offs used in the current clinical practice and in previous studies.^21^, ^23^

### 2.6 Acquisition and processing of structural brain MRI scans

All eligible MRI participants were scanned on the 1.5T system (Philips Intera), and the same scanner and sequences were used for baseline and follow‐up MRI examinations.^24^ The core MRI sequences included a 3D axial magnetization‐prepared rapid acquisition gradient echo T1‐weighted sequence (resolution: 0.94 mm × 0.94 mm × 1.5 mm; no gap; repetition time, 15 ms; echo time, 7 ms; flip angle, 15°), a proton‐density/T2‐weighted sequence (resolution: 0.98 mm × 0.98 mm × 3 mm; no gap; repetition time, 3995 ms; echo time, 18/90 ms; echo‐train length, 6; flip angle, 90°), and a fluid‐attenuated inversion recovery (FLAIR) sequence (resolution: 0.90 mm × 0.90 mm × 6 mm; gap between slices: 1 mm; repetition time, 6000 ms; echo time, 100 ms; inversion time, 1900 ms; echo‐train length, 21; flip angle 90°).

As previously reported, we assessed imaging markers of pathological brain aging on structural brain MRI scans.^24^ In brief, a senior neuroimaging analyst (G.K.) drew WMHs on FLAIR images and further interpolated them on the corresponding T1‐weighted images to compensate for the gap between slices using MRIcron (https://www.nitrc.org/projects/mricron). Then, the global WMH volume was automatically estimated in MRIcron. The inter‐rater reliability was high (> 0.987), and Dice coefficient (which takes into account the spatial overlap of selected hyperintense voxels between the two tracing occasions on a randomly selected subset of 30 FLAIR images) was on average 0.76.^25^ T1‐weighted images were segmented into gray matter, white matter, and cerebrospinal fluid volumes using Statistical Parametric Mapping software in MATLAB (https://www.fil.ion.ucl.ac.uk/spm/). Total intracranial volume (TIV) was obtained by summing gray matter, white matter, and cerebrospinal fluid volume. Hippocampal volume was extracted via an automated segmentation of the T1‐weighted images using the FreeSurfer 5.1 image analysis suite, and the lateral ventricular volume was estimated via an automated segmentation performed with the ALVIN toolbox.^26^ All brain volumes were adjusted for TIV.^27^ The WMH volume was log‐transformed due to the right‐skewed distribution.

A trained rater (Y.L.) visually assessed the presence of lacunes (yes/no) at baseline and follow‐ups, using FLAIR sequence, according to the Standards for Reporting Vascular Changes on Neuroimaging criteria.^28^ We defined prevalent lacunes as the presence of any lacunes at baseline and new lacunes as any lacune emerging during follow‐up periods.^24^ Lacunes were tracked over time and only new lacunes that appeared at a different location have been recorded as incident lacunes.^24^

### 2.7 Statistical analyses

Cox proportional hazard models were used to estimate hazard ratios (HRs) and 95% confidence intervals (CIs) of dementia, CIND, and progression from CIND to dementia in relation to PEF values at baseline. Individuals were considered at risk until their CIND or dementia diagnosis, death, or end of follow‐up, whichever came first. The proportional hazard assumption was assessed by regressing the scaled Schoenfeld residuals against survival time. No deviation from proportionality was detected. Because both the exposure and the outcome of the study are strongly influenced by age, age was used as timescale in the survival analyses.

Potential confounders were defined a priori and chosen based on the literature review and available data from the study population. We reported the main results from three models: Model 1 was adjusted for age, sex, and education; Model 2 was additionally adjusted for smoking, COPD, asthma, use of inhaled bronchodilators, and the number of chronic diseases; and Model 3 was further adjusted for gait speed and undernutrition.

### 2.8 Sensitivity analyses

The following sensitivity analyses have been conducted: (1) When dementia was analyzed as an outcome, we repeated the analyses by excluding those who developed dementia within the first 3 years of follow‐up to assess the potential impact of reverse causation. (2) Because smoking is a strong confounder in the studied association, we have repeated the analyses including only individuals who have never smoked. (3) We have further adjusted the analyses for cardio‐ and cerebrovascular conditions.

In the brain MRI subsample, we used linear mixed‐effects models to estimate the association between PEF at baseline and the levels (intercept) and rates of change (slope) in brain volumes (i.e., total brain tissue volume, hippocampal volume, lateral ventricular volume, and WMH volume) with random participant‐specific intercepts and random slopes for follow‐up time. The interaction terms between follow‐up time and PEF were included as a fixed effect. Log‐binomial regression models were used to estimate the odds ratio and 95% CIs for prevalent lacunes or any new lacune during follow‐up in relation to PEF values at baseline. Models were adjusted for age, sex, education, smoking, COPD, asthma, use of inhaled bronchodilators, number of chronic diseases, gait speed, and undernutrition.

All statistical analyses were performed with Stata Statistical Software: Release 17 (StataCorp LLC).

## 3 RESULTS

Baseline characteristics of study participants in the analytic samples by cognitive status (dementia free, cognitively intact, and CIND) and PEF levels are reported in Table 1. The mean age of the 2323 baseline dementia‐free participants was 71.8 years (SD, 9.5 years), and 60.6% were women.

**TABLE 1:** Baseline characteristics of dementia free (*N* = 2323), cognitively intact (*N* = 1471), and CIND (*N* = 420) analytical samples by peak expiratory flow (PEF) expressed as standard residual percentiles.

	Dementia‐free analytical sample (*N* = 2323)				Cognitively intact analytical sample (*N* = 1471)				CIND analytical sample (*N* = 420)			
Characteristics	<10th (*n* = 276)	10th to 49th (*n* = 923)	50th to 79th (*n* = 818)	> = 80th (*n* = 306)	<10th (*n* = 123)	10th to 49th (*n* = 565)	50th to 79th (*n* = 559)	> = 80th (*n* = 224)	<10th (*n* = 73)	10th to 49th (*n* = 127)	50th to 79th (*n* = 177)	> = 80th (*n* = 73)
Age (years), mean (SD)	73.2 (9.7)	71.0 (9.5)	71.1 (9.2)	74.6 (9.8)	72.1 (9.8)	69.5 (8.9)	69.7 (8.7)	73.9 (10.1)	71.8 (8.5)	70.4 (8.6)	73.1 (9.2)	74.8 (7.7)
Sex (female)	162 (58.7)	541 (58.6)	504 (61.6)	202 (66.0)	71 (57.7)	328 (58.1)	344 (61.5)	142 (63.4)	41 (56.2)	116 (65.5)	93 (73.2)	27 (62.8)
Education (university level)	81 (29.4)	356 (38.6)	344 (42.1)	107 (35.0)	51 (41.5)	247 (43.7)	268 (47.9)	90 (40.2)	15 (20.6)	51 (28.8)	35 (27.6)	5 (11.6)
Ever smoking	91 (33.0)	397 (43.0)	394 (48.2)	160 (52.3)	47 (38.2)	246 (43.5)	259 (46.3)	112 (50.0)	19 (26.0)	75 (42.4)	67 (52.8)	26 (60.5)
Undernutrition^a^	18 (6.5)	23 (2.5)	7 (0.9)	2 (0.6)	6 (4.9)	11 (2.0)	4 (0.7)	0 (0.0)	3 (4.1)	4 (2.3)	2 (1.6)	1 (2.3)
No. of chronic diseases, mean (SD)	4.2 (2.5)	3.8 (2.3)	3.4 (2.2)	3.7 (2.1)	3.8 (2.2)	3.5 (2.2)	3.2 (2.1)	3.5 (2.0)	4.4 (2.7)	3.7 (2.2)	3.8 (2.1)	3.7 (1.9)
COPD	44 (15.9)	44 (4.8)	17 (2.1)	5 (1.6)	16 (13.0)	24 (4.3)	14 (2.5)	4 (1.8)	12 (16.4)	7 (3.9)	1 (0.8)	1 (2.3)
Asthma	28 (10.1)	62 (6.7)	43 (5.3)	10 (3.3)	12 (9.8)	44 (7.8)	33 (5.9)	8 (3.6)	7 (10.0)	12 (6.8)	7 (5.5)	1 (2.3)
Use of inhalators	35 (12.7)	86 (9.3)	62 (7.6)	15 (4.9)	20 (16.3)	59 (10.4)	46 (8.2)	10 (4.5)	7 (9.6)	13 (7.3)	8 (6.3)	2 (4.7)
Walking speed (m/s), mean (SD)	0.9 (0.4)	1.0 (0.4)	1.1 (0.3)	1.2 (0.3)	1.0 (0.4)	1.1 (0.3)	1.2 (0.3)	1.2 (0.3)	0.9 (0.3)	1.0 (0.4)	1.1 (0.3)	1.1 (0.3)
MMSE, mean (SD)	29.0 (1.0)	29.1 (0.9)	29.2 (0.8)	29.1 (0.9)	29.1 (0.9)	29.3 (0.8)	29.3 (0.8)	29.3 (0.8)	28.9 (1.0)	28.9 (0.9)	28.9 (1.0)	28.7 (0.9)

*Note*: Data are expressed as *n* (%) unless otherwise specified. Missing data in the dementia‐free cohort accounted for 0.04% (*N* = 1) in education, 0.43% (*N* = 10) in smoking, and 0.17% (*N* = 4) in MMSE score. Missing data in the cognitively intact cohort accounted for 0.48% (*N* = 7) in smoking. Missing data in the CIND cohort accounted for 0.48% (*N* = 2) in smoking.

Abbreviations: CIND, cognitive impairment, no dementia; COPD, chronic obstructive pulmonary disease; MMSE, Mini‐Mental State Examination; SD, standard deviation.

^a^ Undernutrition was defined as body mass index <18.5 kg/m^2^.

During an average 9‐year follow‐up (mean per person: 9.41 years; SD: 3.49 years), 316 incident dementia cases were identified in the baseline dementia‐free cohort. Of these, 191 were identified via SNAC‐K follow‐up assessments, and an additional 125 dementia cases were captured among those who died, through the medical charts and cause of death register.

### 3.1 Association of lung function with incident dementia

Table 2 presents the association of PEF levels, expressed as SR percentiles, with incident dementia, in the baseline dementia‐free analytical sample. Compared to people with PEF levels ≥ 80th percentile, those belonging to the lowest percentile (< 10th) had a significant 2.25‐fold increased hazard of dementia in a basic‐adjusted model (Model 1). The association did not change when we further adjusted for COPD, asthma, number of chronic diseases, and use of bronchodilators (Model 2), and was similar but slightly attenuated when baseline gait speed and undernutrition were further included in the model (Model 3: HR = 2.02; 95% CI: 1.33–3.08).

**TABLE 2:** Adjusted hazard ratios of dementia with 95% confidence intervals by baseline PEF in the dementia‐free analytical sample (*N* = 2323).

PEF expressed as SR percentiles	No. of	No. of dementia cases	Hazard ratio (95% confidence interval)		
	participants		Model 1	Model 2	Model 3
**Continuous SR PEF** (per 10th SR percentile decrease)			1.08 (1.04–1.12)	1.07 (1.03–1.12)	1.06 (1.02–1.11)
**PEF SR, percentiles**					
≥ 80th	306	50	1.00 (Reference)	1.00 (Reference)	1.00 (Reference)
50th to 79th	818	102	1.14 (00.81–1.61)	1.15 (0.82–1.61)	1.13 (0.80–1.60)
10th to 49th	923	112	1.35 (0.97–1.89)	1.33 (0.95–1.87)	1.33 (0.94–1.88)
< 10th	276	52	**2.25** **(1.52–3.33)**	**2.21** **(1.49–3.29)**	**2.02** **(1.33–3.08)**
*P* for trend			**<0.001**	**<0.001**	**0.001**

*Note*: Hazard ratio (95% confidence interval) was derived from Cox models using age as time scale. Model 1 was adjusted for sex and education; Model 2 was additionally adjusted for the number of chronic diseases, chronic obstructive pulmonary disease, asthma, smoking, and use of bronchodilators; and in Model 3, undernutrition and walking speed were added to Model 2.

Abbreviations: PEF, peak expiratory flow; SR, standardized residual.

Bold values significance *p* < 0.05.

In the sensitivity analysis, we repeated the analysis by excluding individuals who developed dementia within the first 3 years, which yielded similar results (in the fully adjusted model: HR for PEF level as SR of 50 to 79th percentile: 1.69; 95% CI: 1.04–2.77; for PEF as SR 10 to 49th percentile: 1.85; 1.04–3.08; and for PEF as SR < 10th percentile: 2.76; 1.65–5.79).

### 3.2 Association of lung function with incident CIND

During an average period of 9 years of follow‐up (mean per person: 9.69; SD: 3.88 years), 378 incident CIND cases were identified in the baseline cognitively intact analytical sample. Table 3 reports the association of PEF levels with CIND in dementia‐ and CIND‐free participants at baseline. The lowest PEF percentile (< 10th vs. ≥ 80th percentile) was associated with a 55% increased hazard for CIND in the demographic‐adjusted model (Model 1). The observed association remained similar in Model 2 (HR = 1.49; 95% CI: 0.99–2.27), and Model 3 (HR = 1.52; 95% CI: 0.99–2.33).

**TABLE 3:** Adjusted hazard ratios of CIND with 95% confidence intervals by baseline PEF in the cognitively intact analytical sample (*N* = 1471).

PEF expressed as SR percentiles	No. of	No. of dementia cases	Hazard ratio (95% CIs)		
	participants		Model 1	Model 2	Model 3
**Continuous SR PEF** (per 10th SR percentile decrease)			1.05 (1.01–1.09)	1.05 (1.01–1.09)	1.05 (1.01–1.09)
**PEF SR, percentiles**					
≥ 80th	306	50	1.00 (Reference)	1.00 (Reference)	1.00 (Reference)
50th to 79th	818	102	1.05 (0.76–1.44)	1.03 (0.75–1.42)	1.05 (0.76–1.46)
10th to 49th	923	112	1.31 (0.96–1.79)	1.31 (0.95–1.42)	1.33 (0.97–1.84)
< 10th	276	52	**1.55** **(1.03–2.35)**	**1.49** **(0.99–2.27)**	**1.52** **(0.99–2.33)**
*P* for trend			**0.008**	**0.012**	**0.011**

*Note*: Hazard ratio (95% CI) was derived from Cox models using age as timescale. Model 1 was adjusted for sex and education; Model 2 was additionally adjusted for the number of chronic diseases, chronic obstructive pulmonary disease, asthma, smoking, and use of bronchodilators; and in Model 3, undernutrition and walking speed were added to Model 2.

Abbreviations: CI, confidence interval; CIND, cognitive impairment, no dementia; PEF, peak expiratory flow; SR, standardized residual.

Bold values significance *p* < 0.05.

### 3.3 Association of lung function with progression from CIND to dementia

During an average period of 9 years (mean per person: 8.90; SD: 3.60 years), 79 incident dementia cases were identified in the baseline CIND analytical sample. Table 4 reports the HRs for dementia associated with PEF levels in the baseline CIND population. Individuals in the lowest PEF percentile (<10th vs. ≥ 80th percentile) had a 3.39‐fold increased hazard of progressing to dementia in the demographic‐adjusted model. The observed association remained similar when we further adjusted for COPD, asthma, number of chronic diseases, and use of bronchodilators (HR: 3.31 95% CI: 1.26–8.73), and became statistically non‐significant when we adjusted for baseline gait speed and undernutrition (HR: 2.24; 95% CI: 0.80–6.25).

**TABLE 4:** Adjusted hazard ratios of progression from CIND to dementia with 95% CIs by baseline PEF in the CIND analytical sample (*N* = 420).

PEF expressed as SR percentiles	No. of	No. of dementia cases	Hazard ratio (95% CI)		
	participants		Model 1	Model 2	Model 3
**Continuous SR PEF** (per 10th SR percentile decrease)			1.07 (1.00–1.16)	1.07 (0.99–1.16)	1.03 (0.95–1.13)
**PEF SR, percentiles**					
≥ 80th	43	6	1.00 (Reference)	1.00 (Reference)	1.00 (Reference)
50th to 79th	127	29	2.32 (0.95–5.67)	2.23 (0.91–5.48)	1.95 (0.78–4.79)
10th to 49th	177	28	**2.59** **(1.05–6.39)**	**2.51** **(1.02–6.21)**	2.28 (0.91–5.71)
< 10th	73	16	**3.39** **(1.31–8.76)**	**3.31** **(1.26–8.73)**	2.24 (0.80–6.25)
*P* for trend			**0.014**	**0.019**	0.132

*Note*: Hazard ratio (95% CI) was derived from Cox models using age as time scale. Model 1 was adjusted for sex and education; Model 2 was additionally adjusted for the number of chronic diseases, chronic obstructive pulmonary disease, asthma, smoking, and use of bronchodilators; and in Model 3, undernutrition and walking speed were added to Model 2.

Abbreviations: CI, confidence interval; CIND, cognitive impairment, no dementia; PEF, peak expiratory flow; SR, standardized residual.

Bold values significance *p* < 0.05.

#### 3.3.1 Sensitivity analyses

When repeating the analyses including only individuals who have never smoked, the findings remained consistent, although attenuated in the case of dementia incidence. The associations were attenuated and no longer statistically significant for CIND incidence and progression to dementia from CIND (Table S1 in supporting information). Consistent findings have been obtained when we further adjusted the main analyses for cardio‐ and cerebrovascular conditions (Table S2 in supporting information).

### 3.4 Association of lung function with brain MRI markers

The baseline characteristics of study participants in the MRI analytic samples by PEF are reported in Table S3 in supporting information. The mean age of the 462 participants who underwent the MRI scans was 70.9 years (SD, 8.9 years), and 60% were women. Among the 462 MRI participants, the average values for total brain tissue, hippocampal volume, ventricular volume, and WMH volume were 1054.2 mL (SD = 74.3), 7.5 mL (SD = 0.8), 40.6 mL (SD = 18.3), and 1.5 mL (SD = 0.98), respectively. A total of 92 participants had prevalent lacunes at baseline (prevalence: 19.9%).

In the MRI sample, participants who dropped out (*N* = 163; 35.3%) had no significant difference in age (71.9 vs. 70.3 years, *P* = 0.061) or female proportion (55.2% vs. 62.5%, *P* = 0.125), while they had a lower educational level (35.6% vs. 47.2% with university education, *P* = 0.045), and were more likely to suffer from a higher number of chronic diseases at baseline (3.8 vs. 3.4, *P* = 0.037), compared to those who participated to the follow‐up examination(s).

**FIGURE 1:**
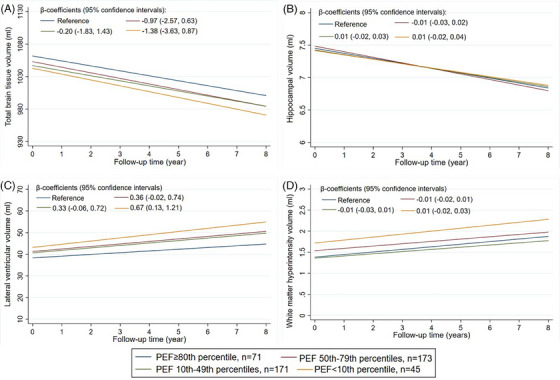
Associations of baseline peak expiratory flow with MRI markers of brain aging. MRI, magnetic resonance imaging; PEF, peak expiratory flow

At baseline, compared to individuals having PEF > 80th percentile, those with PEF values < 10th percentile had on average an 18.94 mL smaller total brain tissue (*β*: −18.94; 95% CI: −38.08, −0.20), and a 0.34 mL greater WMH volume (*β*: 0.34; 95% CI: 0.02–0.65), while they had no difference in the hippocampal volume (*β*: −0.03, 95% CI: −0.27, 0.21) or ventricular volume (*β*: 4.83, 95% CI: −1.17, 10.82). In addition, compared to those in the highest PEF deciles, those in the lowest PEF decile presented a 6.28 times higher likelihood of presenting lacunes at baseline (95% CI: 2.18, 18.85). Figure 1 and Table S4 in supporting information show the trajectories and point estimates with 95% CI of brain volumes over time according to different PEF values at baseline. The rate of ventricular enlargement was 0.67 mL/year faster in people with PEF values below the 10th percentile compared to those having PEF above the 80th percentile (Figure 1). The shrinkage rate of total brain tissue and hippocampus did not differ by different levels of PEF. Table 5 reports the association of different PEF values at baseline with the likelihood of newly developed lacunes after controlling for potential confounders. Compared to people having PEF above the 80th percentile, those with PEF values below the 10th percentile had a 5.05 times higher likelihood of developing lacunes during the follow‐up period

**TABLE 5:** Association of baseline peak expiratory flow with prevalent and incident lacunes (*n* = 460).

	Prevalent lacunes		Incident lacunes	
Peak expiratory flow	*n/N*	Odds ratio (95% confidence interval)	*n/N*	Odds ratio (95% confidence interval)
**Continuous**, per 10th SR percentile decrease	92/460^a^	1.07 (0.97–1.17)	40/296^b^	1.20 (1.04–1.37)^c^
**Percentiles**				
≥ 80th	8/71	1.00 (Reference)	3/50	1.00 (Reference)
50th−79th	40/173	4.05 (1.67–9.82)^d^	14/118	3.69 (0.94–14.49)
10th−49th	28/171	2.39 (0.97–5.93)	8/103	6.39 (1.63–24.97)^d^
< 10th	16/45	6.28 (2.18–18.15)^e^	5/25	5.05 (1.01–25.23)^c^
*P* for trend		0.024		0.012

*Note*: *n/N* indicates the number of cases/number of study participants. Models were derived from log‐binomial regressions adjusted for age, sex, education, the number of chronic diseases, chronic obstructive pulmonary disease, asthma, smoking, use of bronchodilators, undernutrition, and walking speed.

Abbreviations: FLAIR, fluid‐attenuated inversion recovery; MRI, magnetic resonance imaging; SR, standardized residual; WMH, white atter hyperintensity.

^a^ Out of the 462 baseline participants in the MRI subsample, one participant missed information on smoking and one on walking speed.

^b^ Out of the 299 participants with follow‐up images in the MRI subsample, two missed the assessment of lacunes or WMH volumes due to the lack of FLAIR sequences, and one missed information on smoking.

^c^ *P *< 0.05,

^d^ *P *< 0.01,

^e^ *P *< 0.001.

## 4 DISCUSSION

In this population‐based cohort study of Swedish older adults, we examined the associations of lung function as assessed through PEF, with CIND, dementia, and the transition from CIND to dementia, as well as the potential underlying neuropathological mechanisms. The main findings can be summarized into the following two points: (1) Low lung function was associated with an accelerated transition from normal cognition to CIND and dementia in a dose‐dependent manner, and (2) poor lung function was associated with accumulation of cerebral vascular lesions and accelerated global brain atrophy. Taken together, these results suggest that poor pulmonary function is associated with accelerated cognitive deterioration in aging, possibly via mixed neuropathological pathways including cerebral microvascular damage and brain atrophy.

Accumulating evidence supports an association between impaired lung function and cognitive deterioration,^5^, ^29^ including dementia. Two systematic reviews and meta‐analyses of longitudinal studies suggested that every SD decrease in PEF was associated with an ≈ 40% increased risk of dementia,^3^, ^4^ largely in line with our results. While most of the studies point toward an association with dementia, much less is known about the association of lung function with prodromal stages of dementia (e.g., MCI or CIND) and whether impaired lung function accelerates the progression and transition from prodromal dementia to clinical dementia. We here found a 55% increased hazard for CIND associated with PEF values in the lowest percentile, compared to those in the two highest percentiles. In addition, we showed that CIND individuals with the lowest PEF percentile were more likely to progress to dementia. Our results are in line with one recent study of 327 individuals with MCI showing that those with impaired pulmonary function had a 55% increased hazard of developing dementia.^7^ Our study adds further weight to this evidence highlighting that poor pulmonary function is associated with impairment across the whole cognitive continuum, not only with the last dementia stage. In addition, we were able to account for a range of potential confounders in examining these associations.

It is generally recognized that PEF measures the airflow through the bronchi and can reflect the obstructive ventilatory defect in airways.^30^ Previously, data from cohort studies showed that obstructive pulmonary diseases, like COPD and asthma, are associated with late‐life cognitive decline and dementia.^31^, ^32^ Of note, our study showed that the association persisted after adjusting for pulmonary diseases and the use of bronchodilators. This suggests that PEF is linked to cognitive deterioration and transition independently from these conditions. Even if PEF is a rather simple test, good cognitive states are required to understand and perform the task. To account for this aspect, we excluded not only individuals with dementia at baseline but also those with an MMSE < 27, and a strong association of PEF with cognitive outcomes remained. This reinforces the view that PEF might be related to an increased risk for cognitive deterioration. It is worth mentioning that the association between PEF and cognitive outcomes, especially dementia, was somehow attenuated when adjusting for slow gait speed and undernutrition, suggesting that the impact of poor pulmonary function on cognition was partially dependent on frailty. Data from the Rotterdam Study suggested that people with impaired lung function often developed physical frailty with poor reversibility.^33^ This is also in line with our previous study supporting the role of PEF as a marker of general robustness in older adults, with its reduction being associated with frailty development.^10^ Even if not fully explaining the observed association, this is paramount as it suggests that reduced PEF might be a clinical marker of a frail individual at higher risk for future poor cognitive outcomes.

The exact biological mechanisms underlying the association of PEF with cognitive outcomes are not yet fully understood. We explored the longitudinal association between PEF and MRI markers of brain lesions to facilitate the interpretation of the observed PEF–dementia association. The cross‐sectional association of limited expiratory function with lower total brain tissue volume, higher WMH volume, and higher prevalence of lacunes in our study aligns with the reports from previous population‐based studies.^11^, ^13^ In a previous Swedish study involving 379 women aged 70 years and above, lower FEV1 and forced vital capacity (FVC), but not PEF, were associated with WMHs and lacunar infarcts assessed on brain computed tomography scans.^34^ By contrast, we found that low PEF was associated with both prevalent and incident lacunes assessed on brain MRI scans. While the different imaging techniques in assessment of brain lesions make direct comparisons challenging, this disparity may underscore the potential limitation of PEF as an accurate measure of lung function compared to FEV1 and FVC. Interestingly, we found no evidence for a longitudinal association of a lower PEF with volumes of total brain tissue or WMHs, which contrasts with a previous longitudinal study that reported a significant association between poor FEV1 and greater ventricular volume, brain infarcts, and accelerated progression of WMHs.^15^ This further highlights the difference in accuracy between PEF and FEV1 in identifying lung dysfunction and underscores the need for further research. However, one potential explanation could be that ventricular enlargement can reflect the loss of adjacent tissue, particularly in the periventricular region, which is predominantly of white matter and is susceptible to vascular injury.^35^ The absence of a significant association between poor PEF and longitudinal changes in total brain tissue volume suggests that cortical gray matter atrophy may not play a substantial role in this context. This is further supported by the lack of an association between PEF and hippocampal volumes. Future studies are warranted to further assess the association between poor lung function and the hippocampal volume and elucidate its potential role in this association. The association between poor pulmonary function and vascular brain damage found in our study is supported by the evidence that impaired lung function can lead to hypoperfusion and hypoxia with subsequent brain ischemia and atrophy.^3^ Hypoxia may impair both structure and function of the blood–brain barrier and eventually increase the vulnerability for cerebrovascular damage and neuronal necrosis.^36^, ^37^

The major strength of our study refers to the long‐term population‐based prospective cohort study design. In addition, comprehensive clinical and neurocognitive data were integrated with neuroimaging markers for various brain lesions over follow‐up, allowing us to investigate not only the association of lung function with cognitive transitions but also to explore the potential neuropathological mechanisms potentially underlying these associations. Our study also has some limitations. First, some markers of lung function (e.g., FEV1) and brain aging (e.g., cerebral microbleeds and microinfarcts) are not available in our study. Furthermore, because smoking is an important potential confounder in the studied association we both adjusted our main analyses for smoking and repeated the analyses including only individuals who had never smoked. The latter analyses led to attenuated and largely no more statistically significant results. However, it is important to highlight that the point estimates suggested an association between PEF and cognitive outcomes but the exclusion of current and former smokers has resulted in a reduction in sample size and statistical power, which might have contributed to the imprecision of the estimates and the lack of statistical significance. Future studies with larger sample sizes are essential for elucidating the intricate relationship between lung dysfunction and cognitive decline, particularly in individuals who have never smoked. In addition, concerning neuroimaging data, 1.5T scanners may be considered suboptimal according to current standards and may impact the accuracy of ventricular size measurements and the detection of cerebral lacunes. However, MRI data for the current study were collected between 2001 and 2009 when 1.5T scanners were often used in population‐based studies. The relatively small sample size and a short follow‐up period in the MRI sample limited the statistical power, especially in analyzing the longitudinal association of PEF with brain aging markers. Moreover, in SNAC‐K, we conduct a clinical‐based diagnosis of dementia following a three‐step procedure conducted by trained physicians in keeping with the DSM‐IV criteria. Future research including studies with a biological characterization of dementia, and dementia subtypes, is needed to further untangle the relationship between lung function and dementia. Finally, our study sample was derived from central Stockholm, where residents had relatively higher socioeconomic status than the national average. This should be kept in mind when generalizing our study findings to different populations.

In summary, this long‐term population‐based prospective cohort study showed evidence that impaired pulmonary function was associated with an accelerated transition from normal cognition and prodromal dementia to clinical dementia in older adults. Using brain MRI markers, we further revealed that poor lung function is associated with cerebral microvascular damage and accelerated global brain atrophy (ventricular enlargement), which might underlie the association of poor lung function with accelerated cognitive transition from intact cognition through prodromal stages to overt dementia.

## AUTHOR CONTRIBUTIONS

Giulia Grande, Mozhu Ding, Chengxuan Qiu, and Laura Fratiglioni contributed to the conception and design of the study. Giulia Grande and Yuanjing Li conducted the statistical analyses. Giulia Grande and Yuanjing Li conducted the literature search. All the authors contributed to the interpretation of the results. Giulia Grande, Yuanjing Li, and Chengxuan Qiu drafted the first version of the manuscript. All the authors critically revised the manuscript for important intellectual content. All the authors made a significant contribution to the research and the development of the manuscript and approved the final version for publication. SNAC‐K personnel collected the data for the study.

## CONFLICT OF INTEREST STATEMENT

The authors declare no conflicts of interest. Author disclosures are available in the supporting information.

## CONSENT STATEMENT

All participants, or next of kin for cognitively impaired individuals, provided written informed consent.

## Supporting information

Supporting information

Supporting information
