# Supplementary material for: Lung function in relation to brain aging and cognitive transitions in older adults: A population‐based cohort study
Source: Alzheimers Dement. 2024 Jul 5;20(8):5662–73. doi: 10.1002/alz.14079 (PMC11350049; doi:10.1002/alz.14079)
Supplement: Supplementary file 1 — Supporting information [file ALZ-20-5662-s002.docx]

**Supplementary Figure 1.** Flowchart of the study populations

**1471** cognitively intact cohort with complete data

**Aim 2:**

**Incident CIND**

**Aim 1:**

**Incident dementia**

**420** CIND cohort with complete data

Missing data on PEF, N=52

No follow-up data, N=23

**495** participants with CIND at baseline

MMSE <27, N=114

**609** participants with CIND at baseline

Missing data on PEF, N= 129

No follow-up data, N= 308

**1908** dementia and CIND free participants

Dementia at baseline, N=310

CIND/missing information on CIND at baseline, N=1065

MMSE <27, N=80

Missing data on PEF, N=257

No follow-up data, N=165

**2747** dementia-free participants

**3363**

participants at baseline

**Aim 3:**

**Progression from CIND to dementia**

Dementia at baseline, N=310

MMSE <27, N=307

Developmental disorder, N=1

**2323** dementia-free cohort with complete data

**Supplementary Table 1**. Adjusted hazard ratios (HR) of dementia, Cognitive Impairment, no Dementia (CIND) and CIND progression to dementia with 95% confidence intervals by baseline PEF among individuals who have never smoked

| **Baseline PEF expressed as SR percentiles (%)** | **Hazard ratio (95% confidence interval)*** | | |
| --- | --- | --- | --- |
|  | **Incident dementia in baseline dementia-free sample (n=1042)** | **Incident CIND in baseline cognitive intact sample (n=657)** | **Incident dementia in baseline CIND sample (n=183)** |
| ≥80^th^ | 1.00 (Reference) | 1.00 (Reference) | 1.00 (Reference) |
| 50^th^ to 79^th^ | 1.29 (0.79-2.11) | 1.19 (0.75-1.87) | 0.45 (0.12-1.51) |
| 10^th^ to 49^th^ | 1.57 (0.96-2.59) | 1.46 (0.92-2.31) | 0.89 (0.27-2.92) |
| <10^th^ | **2.43 (1.23-4.44)** | 1.36 (0.70-2.64) | 1.00 (0.36-2.41) |

Abbreviations: PEF, peak expiratory flow; CIND, cognitive impairment, no dementia.

*Hazard ratios (95% confidence interval) were derived from Cox models using age as time scale adjusted for sex, education, number of chronic diseases, chronic obstructive pulmonary disease, asthma, use of bronchodilators, undernutrition and walking speed.

**Supplementary Table 2**. Adjusted hazard ratios (HR) of dementia, Cognitive Impairment, no Dementia (CIND) and CIND progression to dementia with 95% confidence intervals by baseline PEF where cardiovascular diseases and diabetes were further adjustedfor

| **Baseline PEF expressed as SR percentiles (%)** | **Hazard ratio (95% confidence interval)*** | | |
| --- | --- | --- | --- |
|  | **Incident dementia in baseline dementia-free sample (n=2262)** | **Incident CIND in baseline cognitive intact sample (n=1448)** | **Incident dementia in baseline CIND sample (n=410)** |
| ≥80^th^ | 1.00 (Reference) | 1.00 (Reference) | 1.00 (Reference) |
| 50^th^ to 79^th^ | 1.14 (0.81-1.61) | 1.05 (0.76-1.45) | 1.95 (0.79-4.83) |
| 10^th^ to 49^th^ | 1.31 (0.92-1.86) | 1.33 (0.96-1.83) | 2.26 (0.90-5.66) |
| <10^th^ | **2.00 (1.32-3.05)** | **1.52 (0.99-2.33)** | 2.24 (0.79-6.21) |

Abbreviations: PEF, peak expiratory flow; CIND, cognitive impairment, no dementia.

*Hazard ratios (95% confidence interval) were derived from Cox models using age as time scale adjusted for sex, education, number of chronic diseases, chronic obstructive pulmonary disease, asthma, smoking, use of bronchodilators, undernutrition, walking speed and cardiovascular diseases (atrial fibrillation, heart failure, ischemic heart disease and cerebrovascular diseases) and diabetes.

**Supplementary Table 3**. Baseline characteristics by peak expiratory flow levels in the cohort with brain MRI measures

| **Characteristics** | **Total** **MRI sample** | **Percentiles of peak expiratory flow** | | | |
| --- | --- | --- | --- | --- | --- |
|  | (n=462) | ≥80^th^ (n=71) | 50^th^ to 79^th^ (n=173) | 10^th^ to 49^th^ (n=173) | <10^th^ (n=45) |
| Age (years), mean (SD) | 70.9 (9.0) | 74.0 (9.7) | 70.3 (8.4) | 69.8 (8.8) | 72.3 (9.3) |
| Female, n (%) | 277 (60.0) | 44 (15.9) | 106 (38.3) | 103 (37.2) | 24 (53.4) |
| University degree, n (%) | 199 (43.1) | 27 (13.6) | 84 (42.2) | 69 (34.7) | 19 (9.6) |
| MMSE, mean (SD) | 29.0 (1.61) | 28.9 (1.24) | 29.1 (2.03) | 29.1 (1.26) | 28.7 (1.43) |
| Number of chronic diseases, mean (SD) | 3.51 (2.11) | 3.76 (2.23) | 3.29 (2.10) | 3.50 (2.09) | 3.98 (1.95) |
| COPD, n (%) | 20 (4.3) | 2 (2.8) | 5 (2.9) | 8 (4.6) | 5 (11.1) |
| Asthma, n (%) | 34 (7.4) | 5 (7.0) | 10 (5.8) | 14 (8.1) | 5 (11.1) |
| Smoking, n (%) |  |  |  |  |  |
| Never | 207 (44.9) | 34 (47.9) | 83 (48.0) | 76 (44.2) | 14 (31.1) |
| Former | 191 (41.4) | 30 (42.3) | 73 (42.2) | 71 (41.3) | 17 (37.8) |
| Current | 63 (13.7) | 7 (9.9) | 17 (9.8) | 25 (14.5) | 14 (31.1) |
| Use of broncodilatators, n (%) | 52 (11.3) | 5 (7.0) | 19 (11.0) | 21 (12.1) | 7 (15.6) |
| Malnutrition, n (%) | 11 (2.4) | 1 (1.4) | 3 (1.7) | 6 (3.5) | 1 (2.22) |
| Walking speed (m/s), mean (SD) | 1.12 (0.33) | 1.10 (0.33) | 1.16 (0.31) | 1.12 (0.35) | 1.02 (0.33) |

Abbreviations: SD, standard deviation; MMSE: Mini Mental State Examination; COPD: chronic obstructive pulmonary disease.

Missing values: 1 in smoking and 1 in walking speed.

**Supplementary** Table 4. β-coefficient and 95% confidence interval between baseline PEF values and brain measures

| **Peak expiratory flow** | **β-coefficient and 95% confidence interval, annual change in the continuous brain measures** | | | |
| --- | --- | --- | --- | --- |
|  | **Total brain tissue volume** | **Hippocampal volume** | **Lateral ventricular volume** | **White matter hyperintensity volume** |
| **Continuous**, per 10^th^ SR percentile decrease | -0.06  (-0.27, 0.15) | 0.002  (-0.001, 0.006) | 0.05  (0.00, 0.10)^a^ | 0.000  (-0.002, 0.003) |
| **Percentiles** |  |  |  |  |
| ≥80th | 0 (Reference) | 0 (Reference) | 0 (Reference) | 0 (Reference) |
| 50th -79th | -0.97 (-2.57, 0.63) | -0.009 (-0.034, 0.015) | 0.36 (-0.02, 0.74) | -0.006 (-0.024, 0.011) |
| 10th -49th | -0.20 (-1.83, 1.43) | 0.006 (-0.019, 0.031) | 0.33 (-0.06, 0.72) | -0.010 (-0.027, 0.008) |
| <10th | -1.38 (-3.63, 0.87) | 0.009 (-0.025, 0.044) | 0.67 (0.13, 1.21)^a^ | 0.009 (-0.016, 0.034) |
| P for trend | 0.673 | 0.249 | 0.038 | 0.992 |

Models were adjusted for age, sex, education, number of chronic diseases, chronic obstructive pulmonary disease, asthma, smoking, use of bronchodilators, undernutrition, and walking speed.

Out of the 462 participants in the MRI subsample, one participant had a missing value in smoking and one in walking speed.

^a^P<0.05, ^b^P<0.01, ^c^P<0.001.
